# Curcumin decreases Warburg effect in cancer cells by down-regulating pyruvate kinase M2 via mTOR-HIF1α inhibition

**DOI:** 10.1038/s41598-018-25524-3

**Published:** 2018-05-29

**Authors:** Farid Ahmad Siddiqui, Gopinath Prakasam, Shilpi Chattopadhyay, Asad Ur Rehman, Rayees Ahmad Padder, Mohammad Afaque Ansari, Rasha Irshad, Kailash Mangalhara, Rameshwar N. K. Bamezai, Mohammad Husain, Syed Mansoor Ali, Mohammad Askandar Iqbal

**Affiliations:** 1 Department of Biotechnology, Faculty of Natural Sciences, Jamia Millia Islamia (A Central University), Jamia Nagar, New Delhi, 110025 India; 2 School of Life Sciences, Jawaharlal Nehru University, New Mehrauli Road, New Delhi, 110067 India; 3 Department of Toxicology, School of Chemical and Life Sciences, Jamia Hamdard (Deemed University), New Delhi, 110062 India; 4 Department of Biosciences, Faculty of Natural Sciences, Jamia Millia Islamia (A Central University), Jamia Nagar, New Delhi, 110025 India

## Abstract

Warburg effect is an emerging hallmark of cancer cells with pyruvate kinase M2 (PKM2) as its key regulator. Curcumin is an extensively-studied anti-cancer compound, however, its role in affecting cancer metabolism remains poorly understood. Herein, we show that curcumin inhibits glucose uptake and lactate production (Warburg effect) in a variety of cancer cell lines by down-regulating PKM2 expression, via inhibition of mTOR-HIF1α axis. Stable PKM2 silencing revealed that PKM2 is required for Warburg effect and proliferation of cancer cells. PKM2 over-expression abrogated the effects of curcumin, demonstrating that inhibition of Warburg effect by curcumin is PKM2-mediated. High PKM2 expression correlated strongly with poor overall survival in cancer, suggesting the requirement of PKM2 in cancer progression. The study unravels novel PKM2-mediated inhibitory effect of curcumin on metabolic capacities of cancer cells. To the best of our knowledge, this is the first study linking curcumin with PKM2-driven cancer glycolysis, thus, providing new perspectives into the mechanism of its anticancer activity.

## Introduction

Metabolic priorities of cancer cells differ remarkably from normal cells, thus providing a new therapeutic window. Metabolic reprogramming in cancer cells support their growth, survival, proliferation and maintenance^1^. In 1920’s, Otto Warburg observed that tumor cells produce large quantities of lactate even when sufficient oxygen is present, a phenomenon referred to as *Warburg effect* or *aerobic glycolysis*^2^. Warburg effect is characterized by high glucose uptake and lactate release and is now considered as a hallmark of nearly all tumors^3^. This metabolic adaptation benefits cancer cells in surviving through hypoxic conditions, commonly found in tumors, and to support their anabolic requirements^4,5^. Tendency of cancer cells to take-up large quantities of glucose is exploited in clinical detection of tumors by 18fluorodeoxyglucose-positron emission tomography (FDG-PET) scan^6^. Hyper-activating mutations in growth signaling are known to induce expression of enzymes that are vital for cancer metabolism^7^. Interest in studying cancer metabolism has been rekindled recently with a burst in number of publications in last decade^8^. Now, metabolism of cancer cells is considered a therapeutic hotspot for dietary and pharmacologic interventions^9–11^.

Extensive studies have shown that pyruvate kinase M2 (PKM2) is one of the critical regulator of Warburg effect^12,13^. Accordingly, PKM2 is highly expressed in proliferating cells like tumor and embryonic cells^14^. PKM2 is one of the four isoform of pyruvate kinase- PKL, PKR, PKM1 and PKM2^15^. Switch towards PKM2 isoform is a pre-requisite for aerobic glycolysis to take place^16^. Oncogenic transcription factor c-Myc has been demonstrated to control the mutually exclusive alternative splicing of PKM mRNA in favor of PKM2^17^. PKM2 expression has been used as a tumor marker^18–21^. We and others have previously reported that PKM2 is associated with tumor metabolism and growth^22,23^. Several studies have suggested PKM2 as a therapeutic target for cancer treatment^24,25^. Therefore, it is pertinent to evaluate drugs that could suppress PKM2 expression, thus, inhibiting cancer metabolism.

Curcumin (diferuloylmethane) is a well-known phytopolyphenolic compound isolated from rhizome of the plant *Curcuma longa* (Zingiberaceae)^26^. Curcumin is considered as a valuable medicinal plant in Indian systems of medicine. Numerous studies have shown the anti-cancer properties of curcumin in a wide variety of cell lines and animals^27–33^. The major features of carcinogenesis have been shown to be inhibited by curcumin^34^. Several mechanisms for anti-cancer activities of curcumin have been proposed, including, induction of apoptosis^34^, p53 stabilization^35^, mTOR^33^, Wnt^36^, Notch^37^, PI3K^38^, signaling inhibition, AMPK activation^39^, cell cycle inhibition^40^, inhibition of oncogenes^41^, inactivation of NF-kB^42^, metastasis inhibition^43^, angiogenesis inhibition^44^, miRNA regulation^45^, DNA damage and repair^46^. However, the effect of curcumin on cancer metabolism, an emerging hallmark of cancer, remains unknown.

Here, we investigated the effect of curcumin on cancer metabolism and report novel PKM2-mediated inhibitory effects of curcumin on Warburg effect. Our results identify a new anti-cancer mechanism of curcumin and endorse its therapeutic relevance in inhibiting cancer.

## Results

### Curcumin inhibits Warburg effect in cancer cells

**Figure 1:**
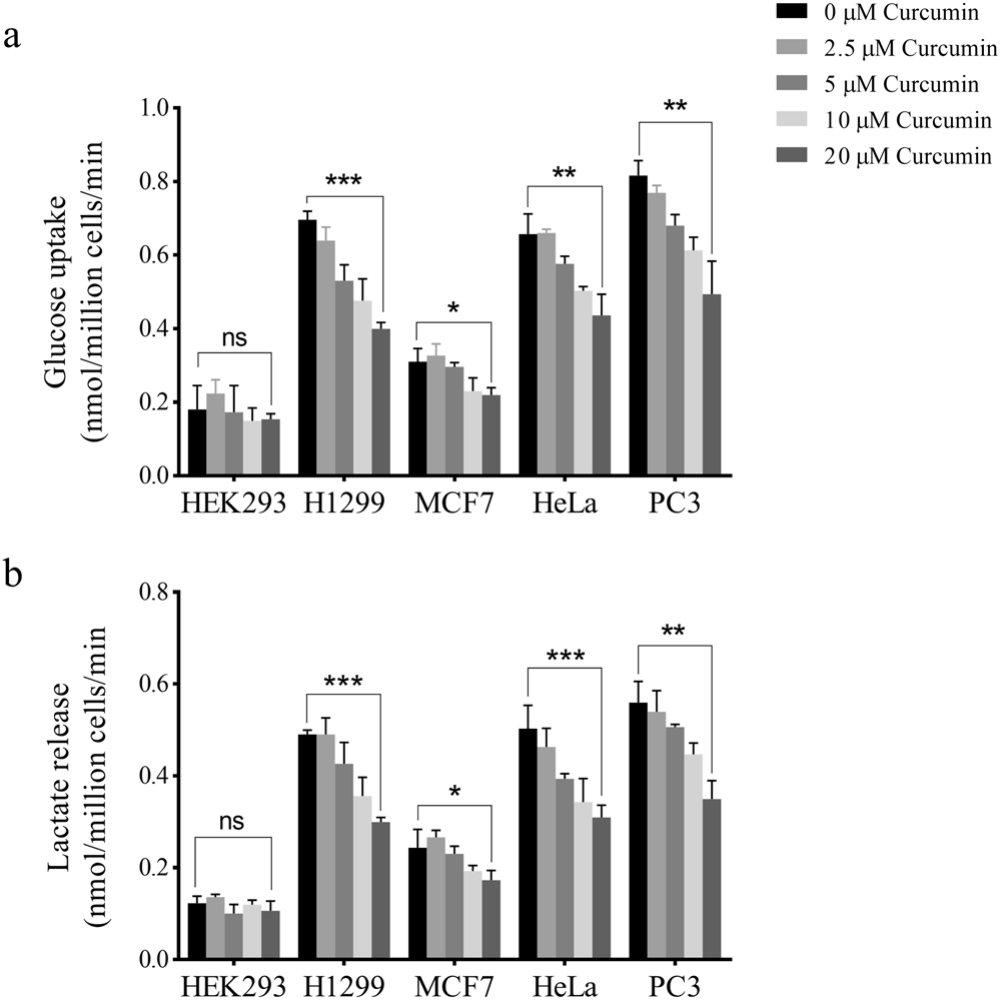
Dose-dependent effect of curcumin on Warburg effect. High glucose uptake and lactate production, also referred to as Warburg effect, is a hallmark feature of cancer cells (see text) needed to support proliferation of cancer cells. Glucose uptake (**a**) and lactate release (**b**) by H1299, MCF-7, HeLa and PC3 cells was reduced significantly upon curcumin treatment compared with HEK293 where the change in glucose and lactate was not significant. Different doses of curcumin (2.5, 5, 10 and 20 μM) for 24 hours were used for treatment purpose. Maximal decrease in Warburg effect was observed at 20 μM. Error bars represent mean ± SD.

The effect of curcumin on Warburg effect was studied by measuring the rate of glucose uptake and lactate production in cancer cell lines- lung (H1299), breast (MCF-7), cervical (HeLa) and prostate (PC3) and human embryonic kidney (HEK) 293 cells, taken as control. Sub-toxic concentrations of 0–20 μM curcumin for 24 hours were used for the study. Significant inhibition in glucose uptake and lactate release was observed across the four cell lines, however, no appreciable decrease in Warburg effect was observed in HEK 293 cells (Fig. 1a,b). Dose-dependent decrease in Warburg effect started at 2.5 μM with maximal decrease at 20 μM curcumin.

### Curcumin down-regulates PKM2 via inhibition of mTOR-HIF1α axis

**Figure 2:**
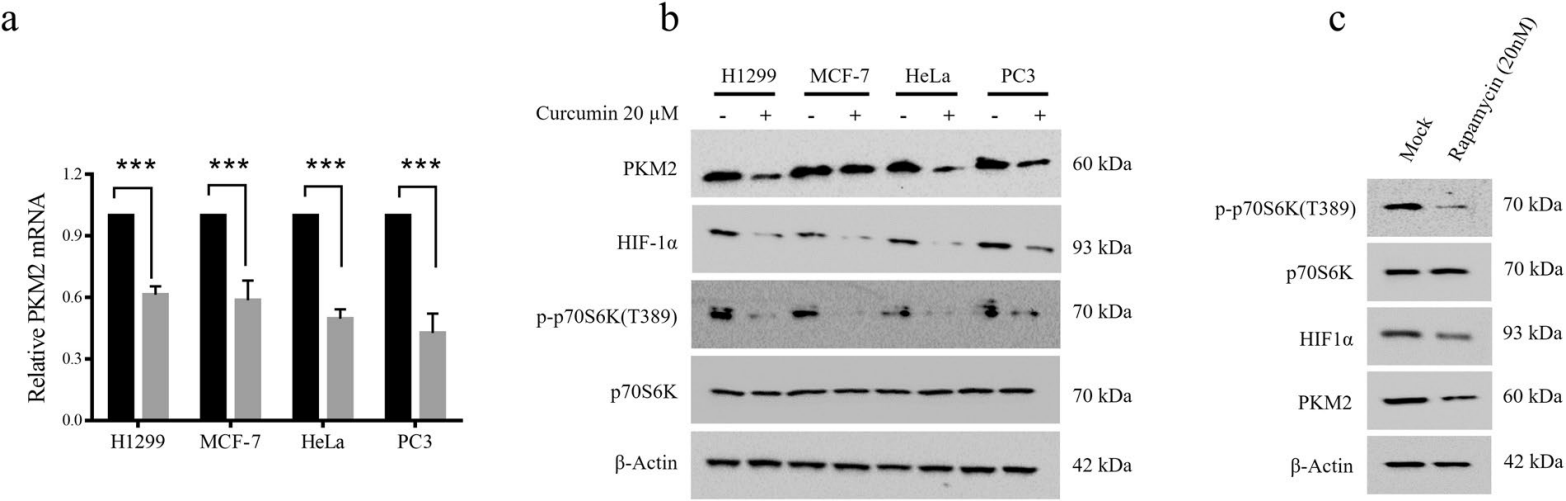
PKM2 expression is inhibited by curcumin through mTOR/HIF1α axis. (**a**) Substantial reduction in PKM2 mRNA in H1299, MCF-7, HeLa and PC3 cells treated with 20 μM for 24 hours. (**b**) Immunoblot of H1299, MCF-7, HeLa and PC3 cells showing coincided reduction in PKM2, HIF1α and phosphorylated-p70S6K (T389), upon curcumin treatment. (**c**) Treatment with standard mTOR inhibitor-rapamycin also showed similar expression pattern. Results suggested that curcumin inhibited PKM2 expression via inhibition of mTOR and HIF1α. Error bars in PKM2 mRNA graph represent mean ± SD.

To understand the decrease in glucose consumption and lactate production by curcumin-treated cell lines, we studied the status of PKM2, a critical regulator of Warburg effect. Since maximal decrease in Warburg effect was observed at 20 μM curcumin, we used this concentration to study the effect of curcumin on PKM2 status in H1299, MCF-7, HeLa and PC3 cell lines. Curcumin treatment substantially reduced PKM2 mRNA and protein as assessed by qRT-PCR and immunoblotting (Fig. 2a,b and Supplementary Figure S2). Further, in an attempt to elucidate the mechanism responsible for PKM2 down-regulation, we studied the mTOR/HIF1α pathway inhibition upon curcumin treatment. mTOR is frequently hyper-activated in various cancers^47^ and curcumin has been shown to inhibit mTOR signaling^48^. HIF1α is a known transcriptional activator of PKM2^13,49^. Upon curcumin treatment, decreased PKM2 expression coincided with decreased Threonine 389 (T389) phosphorylation of p70S6 kinase and decreased HIF1α protein, suggesting that curcumin down regulated PKM2 by inhibiting the mTOR/HIF1α signaling. In addition, inhibition of PKM2 expression by rapamycin (a well-known mTOR inhibitor), further validated that curcumin decreased PKM2 via inhibition of mTOR/HIF1α signaling (Fig. 2c). GLUT1 and HKII mRNA were also found to be decreased upon curcumin treatment, suggesting the contribution of these enzymes, in addition to PKM2, in inhibition of Warburg effect upon curcumin treatment (Supplementary Figure S1).

### Curcumin decreases viability of cancer cells

**Figure 3:**
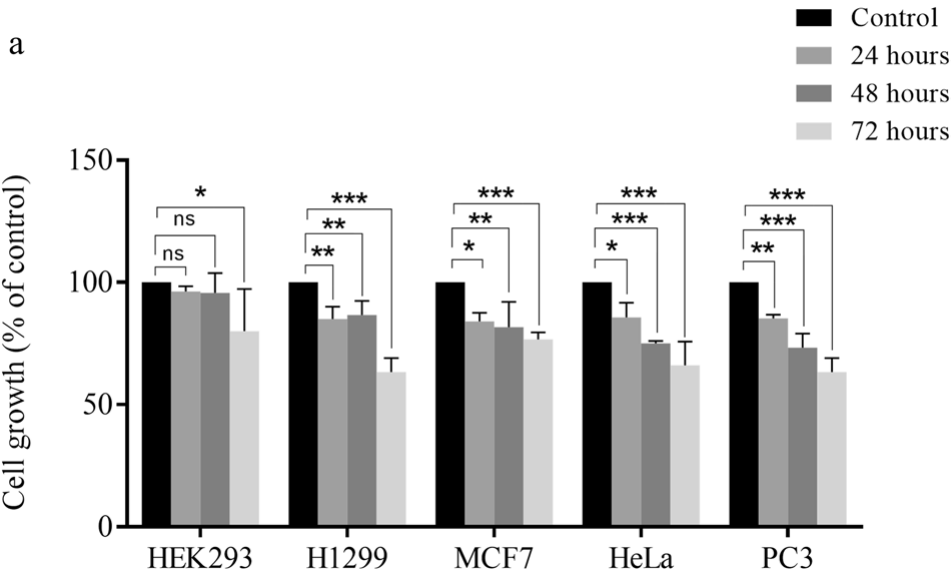
Decreased cell viability upon curcumin treatment. (**a**) Curcumin treatment decreased viability of H1299, MCF-7, HeLa and PC3 cells over the time course of 24, 48 and 72 hours. Decrease in viability of four cancer cell lines is significantly higher compared to control HEK293 cells. Maximum decrease in viability of all studied cell lines was observed at 72 hours. Error bars represent mean ± SD.

To analyze if inhibition of PKM2 and cancer metabolism by curcumin contributed to reduction in viability, growth of HEK293, H1299, MCF-7, HeLa and PC3 cells was assessed over the period of 24–72 hours in presence of 20 μM curcumin. Decreasing trend of viability was observed in a time dependent manner (Fig. 3a). Maximum decrease in viability of all cell lines was observed at 72 hours, although; drop in viability started at 24 hours. However, no significant decrease in viability was observed in control HEK 293 cells.

### PKM2 silencing decreases Warburg effect and cell viability

**Figure 4:**
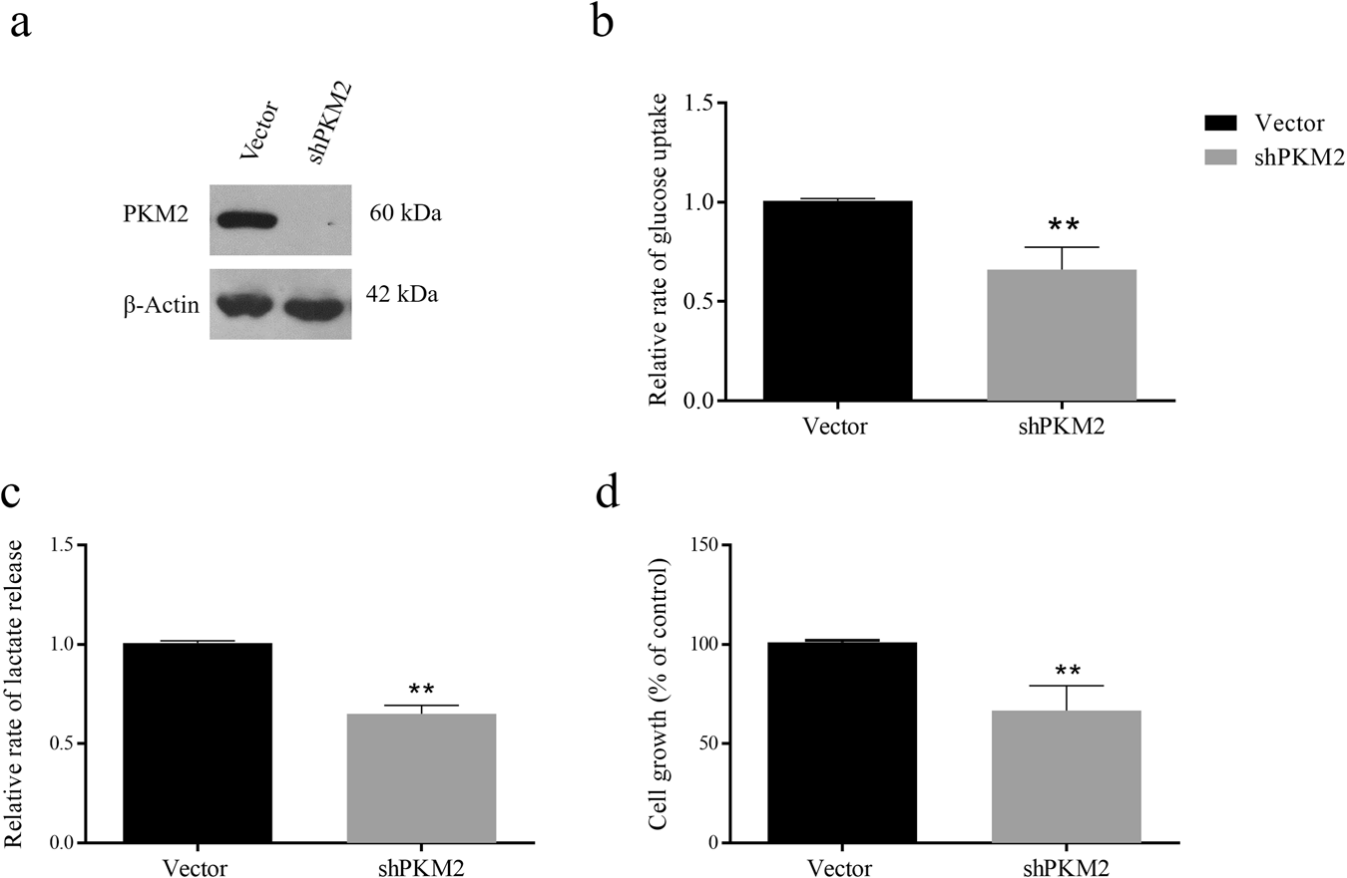
Knock-down of PKM2 reduces glucose uptake, lactate production and cell viability. PKM2 was stably silenced in H1299 cells using lentiviral shRNA as described in methods. (**a**) Confirmation of PKM2 knockdown by Western blotting, (**b**) drop in glucose uptake, (**c**) lactate production and (**d**) cell viability was observed. Error bars in PKM2 mRNA graph represent mean ± SD.

We conjectured that the decrease in Warburg effect upon curcumin treatment is, at least in part, due to down-regulation of PKM2 expression. To this end, PKM2 was stably silenced in H1299 cells using shRNA approach. Knock-down efficiency was checked by Western blotting (Fig. 4a). Thereafter, glucose consumption and lactate release were measured. shPKM2 transfected H1299 cells exhibited significant reduction in consumption of glucose and lactate production, indicating that PKM2 is crucial for Warburg effect (Fig. 4b,c). These results validated that curcumin inhibited aerobic glycolysis by down-regulating PKM2 expression. In addition to Warburg effect, PKM2 silencing also reduced viability of H1299 cells (Fig. 4d), further suggesting that PKM2-driven Warburg effect is essential for survival of cancer cells.

### Over-expressing PKM2 reverses the effect of curcumin on metabolism of cancer cells

**Figure 5:**
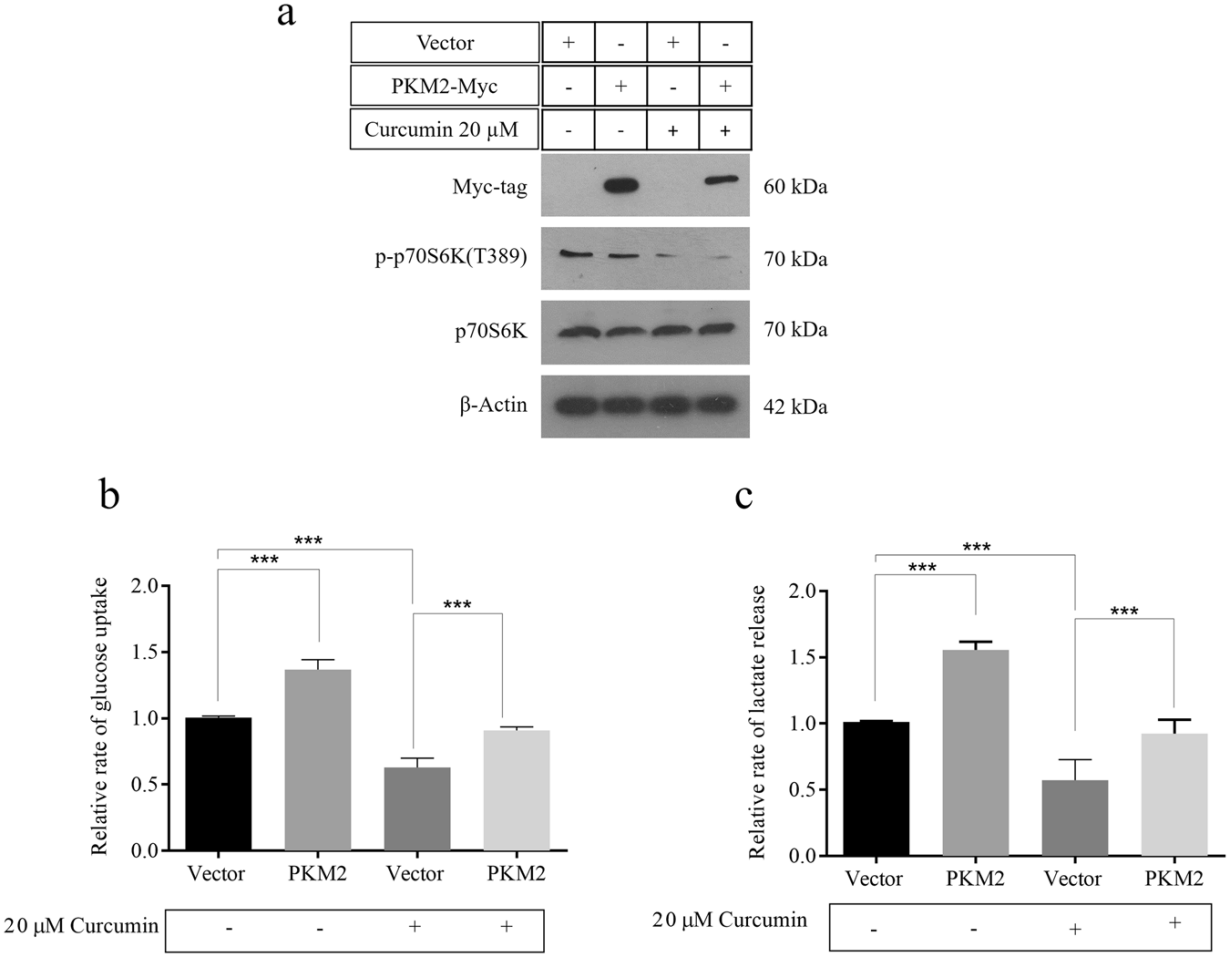
PKM2 over-expression reversed the effects of curcumin on cancer glycolysis. (**a**) Immunoblot confirming myc-PKM2 over-expression in H1299 cells continually exposed to 20 μM curcumin. Nearly diminished p-p70SK (T389) in both vector and PKM2 transfected H1299 cells confirmed mTOR inhibition by curcumin. Increased glucose uptake (**b**) and lactate release (**c**) in H1299 cells over-expressing PKM2, compared to vector transfected, in absence and presence of curcumin.

In order to further validate that inhibitory effects of curcumin on cancer glycolysis are PKM2-mediated, PKM2 was transiently over-expressed in H1299 cells in absence and presence of 20 μM curcumin. H1299 cells were either transfected with vector control or myc-PKM2. Confirmation of transfection was done by immunoblotting using anti-myc antibodies (Fig. 5a). Curcumin activity was measured by checking the inhibition of p70S6 kinase T389 phosphorylation. Expectedly, PKM2 over-expression resulted in augmented Warburg effect even in continuous presence of 20 μM curcumin (Fig. 5b,c). Results demonstrated the ectopic PKM2 expression repressed the effects of curcumin on glucose uptake and lactate release, substantiating that PKM2 is a target of curcumin. In absence of curcumin, PKM2 transfected cells compared with vector showed the expected increase in glucose uptake and lactate production. Consistent with Fig. 1, treated vector control showed decreased glucose and lactate compared with untreated vector control.

### PKM2 expression is higher in cancer and associated with poor overall survival

**Figure 6:**
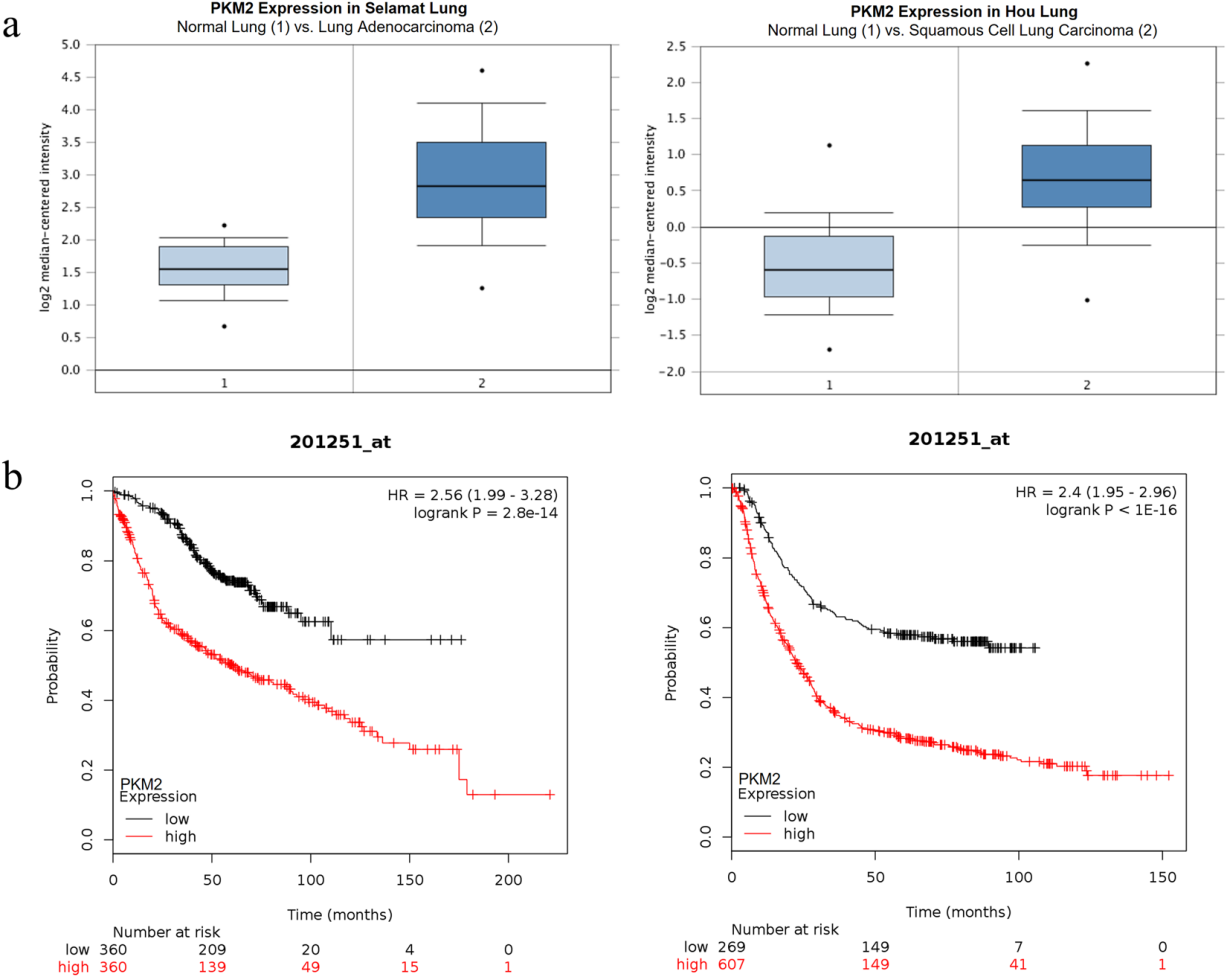
PKM2 expression is high in cancer and correlates with poor overall survival. (**a**) Box plots from Oncomine representing the higher PKM2 expression in lung adenocarcinoma and squamous cell lung carcinoma, compared to normal lung. (**b**) Kaplan-Meier overall survival (OS) curve of patients with lung cancer expressing low and high PKM2 mRNA. (PKM2 low expression group, n = 360; PKM2 high expression group, n = 360) and Kaplan-Meier overall survival (OS) curve of patients with gastric cancer expressing low and high PKM2 mRNA. (PKM2 low expression group, n = 269; PKM2 high expression group, n = 607).

To investigate the expression levels of PKM2 in tumour tissues, Oncomine database was utilised. In Selamat lung cancer study comparing expression of PKM2 in normal lung (n = 58) with the lung adenocarcinoma tissue (n = 58), PKM2 expression was found to be 2.5-fold higher in lung adenocarcinoma compared to normal lung (Fig. 6a)^50^. Similarly, Hou dataset also revealed 2.3-fold increase in PKM2 expression in squamous cell lung carcinoma as compared with normal lung (n = 65) (Fig. 6a)^51^. Further, we performed Kaplan-Meier analysis using online portal (www.kmplot.com) to correlate PKM2 expression with overall survival of cancer patients. Higher PKM2 expression correlated strongly with poor overall survival in lung and gastric cancer patients (Fig. 6b)^52,53^. These results suggest that higher PKM2 expression is associated with cancer and may represent a useful prognostic marker.

## Discussion

Metabolic transformation in cancer cells has gained enormous attention in recent past for its immense potential as viable therapeutic target. Owing to the metabolic vulnerabilities of cancer cells, several drugs that target cancer metabolism are under clinical trials. Just as with any chemotherapeutic drug, drugs that target metabolism of cancer cells may have side-effects that could deteriorate patient’s health and life-quality. On the contrary, natural plant based compounds have relatively less toxicity and side-effects. Therefore, there is an urgent need to screen more natural compounds for their inhibitory effects on the emerging hallmarks of cancer, like metabolism. Till date, very few phytochemicals have been shown to target cancer metabolism. Cleary, there is a need to screen more natural compounds for their negative effects on cancer metabolism. Metabolic enzymes could be therapeutic hotspots, provided they are key regulators of metabolic pathways operating in cancer cells. Large body of evidence suggests that PKM2 is the key regulator of cancer metabolism, thus, targeting PKM2 to inhibit cancer metabolism should be a viable anti-cancer strategy.

Curcumin has been well-studied as a inhibitor of a variety of cancer features; however, its effects on cancer metabolism remained un-elucidated. We demonstrated that curcumin inhibits cancer metabolism in a PKM2-dependent manner. Results presented here unravel a new dimension of anti-cancer mechanisms of curcumin. Results from this work, along with the already known literature on curcumin, suggest that curcumin inhibits most, if not all, hallmarks of cancer, making it a powerful plant-based anti-cancer compound. However, there are several caveats associated with the stability and bioavailability of curcumin, thus, hindering its application in clinical setting^54^. Owing to the unstable chemical nature of curcumin, it is known to degrade within few hours in culture media, thus, making its bioavailability abysmal^55^. Nonetheless, numerous studies have demonstrated the anti-cancer properties of curcumin^56^. This raises the possibility of role of the degradation products of curcumin in contributing to its onco-pharmacological effects^57^.

The effect of curcumin on Warburg effect (Fig. 1) was found to be consistent across all four cancer cell lines studied, highlighting the spectrum of curcumin as an anti-cancer drug. Besides, these results also hint at the near-universal requirement of Warburg effect by solid cancers for their growth and survival. Interestingly, curcumin-induced inhibition of metabolism was evident at a low 2.5 μM concentration, demonstrating the sensitivity of cancer cells to curcumin and dependency on aerobic glycolysis. Notably, the negligible effects of curcumin on glycolysis and viability of epithelial HEK 293 cells suggested that the observed inhibition of Warburg effect by curcumin is specific to cancer cells. The decrease in glucose consumption and lactate production impedes the growth of cancer cells because glycolytic flux provides for anabolic synthesis in cancer cells to produce macromolecules for daughter cancer cells. Furthermore, intermediates of glycolysis acts as precursors or intermediates of cross-talking anabolic pathway like pentose phosphate pathway. Besides, high rates of glycolysis in cancer also serve the purpose of rapid ATP production, as in case of muscle during heavy exercise. Therefore, it is fathomable that high glycolytic rate is a life-line of dividing cancer cells. The decrease in PKM2 expression upon curcumin treatment explains the observed decrease in aerobic glycolysis in cell lines treated with curcumin. Since PKM2 acts a regulator of glycolytic flux^12^, through its protein and pyruvate kinase activity, changes in its expression are expected to cause changes in glycolytic flux. Accordingly, silencing of PKM2 suppressed the Warburg effect and viability (Fig. 3). Moreover, higher PKM2 expression in cancer tissues and statistically significant association with poor overall survival of cancer patients, strongly suggested the role of PKM2 in cancer (Fig. 6).

mTOR is an important growth regulator and is usually very active in cancer cells due to mutations in regulatory pathways or the mTOR itself^58,59^. The role of mTOR in protein synthesis makes it indispensable for the survival of cancer cells. The regulation of HIF1α and PKM2 expression by mTOR highlights the importance of mTOR signaling in regulation of cancer metabolism (Fig. 2). mTOR is a tuner of metabolism because latter provides raw-material for protein synthesis i.e. amino acids and is known to regulate HIF1α and PKM2 expression^23,60,61^. Therefore, inhibition of mTOR is expected to inhibit PKM2 and glycolysis, which otherwise support anabolic metabolism in cancer^62^. Inhibition of Warburg effect results in decreased anabolism and therefore the viability of cancer cells is negatively affected by curcumin (Fig. 3) and silencing of PKM2 (Fig. 4) as all these factors converge to suppress macromolecular synthesis. The role of HIF1α in controlling the expression of PKM2 is an outcome of hypoxic conditions that exists in tumor cells. PKM2 can drive glycolysis even in absence of oxygen, by promoting the conversion of pyruvate to lactate through a largely unknown mechanism, thus, induction of PKM2 by HIF1α is an adaptation necessary for tumor growth. Abrogation of inhibitory effects of curcumin on aerobic glycolysis in PKM2 over-expressing cells not only suggested that effects were mediated by PKM2 but also highlighted the importance of PKM2 for cancer cell metabolism (Fig. 5).

Although curcumin has been shown to exert anti-cancer effects through a variety of mechanisms, it is important to identify mechanisms that could be targeted without harming normal cells. Metabolic rewiring in cancer cells is one such mechanism. The work presented here represents a new anti-cancer mechanism of curcumin and pin-points the enzyme responsible for mediating the anti-cancer effects of curcumin. Since bioavailability of curcumin is a challenge faced in clinical setting; stable analogues of curcumin should be tested for their ability to mimic inhibitory effects of curcumin on cancer metabolism.

In summary, this study unravels a new anti-cancer role of curcumin and invites further research into exploiting curcumin and its analogues for successful clinical inhibition of metabolic addictions in cancer cells.

## Materials and Methods

### Cell culture and drug treatment

HEK293, H1299, MCF-7, HeLa and PC3 cell lines were procured and maintained as described^63^. Briefly, cell lines were grown in monolayer and passaged routinely 2–3 times a week. Cell lines were maintained either in DMEM or RPMI with 10% FBS (Gibco) and 1% penicillin/streptomycin (Sigma) at 37°C and 5% CO_2_ in a humidified incubator. 2 mM Curcumin (Sigma, MO, USA) and 200 µM rapamycin (Sigma, MO, USA) stock solutions were prepared using DMSO and stored at −80°C, until further use. Prior to drug treatment, cells were allowed to grow for 24 hours followed by treatment with either DMSO (mock control) or curcumin for 24–72 hours.

### Metabolic assays

For glucose and lactate: Spent media was collected, centrifuged to remove any cell debris and deproteinized using 5% trichloroacetic acid (TCA) and glucose and lactate were assayed using colorimetric kits (BioVision, USA) as per the manufacturer’s instructions. Standard curves were prepared and background corrections were done. All measurements were normalized to cell numbers. Rate of glucose uptake and lactate production were measured and expressed as nmol/million cells/minute.

### Western blotting and qRT-PCR

Whole cell lysate was prepared in modified RIPA buffer containing 50 mM Tris-Cl pH 7.2, 150 mM NaCl, 0.5% sodium deoxycholate, 0.1% SDS, 1% Triton X-100 with added 1 mM dithiothreitol (DTT), 1 mM phenylmethylsulfonyl fluoride (PMSF), protease and phosphatse inhibitor cocktail (Geno Biosciences, USA). Lysates were then centrifuged and supernatant containing protein was quantified using BCA (bicinchoninic acid) kit (Thermo Scientific, USA) as per the manufacturer protocol. Equal amount of protein was loaded and separated by SDS-PAGE, transferred to nitrocellulose membrane (mdi, USA) and probed with relevant primary antibodies. Membrane was incubated with appropriate secondary antibody for 1 hour at room temperature and proteins were detected using Luminata Forte (Millipore, USA). Densitometry analysis was carried out using ImageJ software to examine the relative gene expression change following normalizing with β-actin. Primary antibodies used were: anti-PKM2, anti-HIF1α, anti-myc, anti-p-p70S6K (T389), anti-p70S6K and anti-β-actin (Cell signaling Technology, USA). For qRT-PCR: total RNA was extracted from cell lines using TRI reagent (Sigma, MO, USA) followed by reverse transcription to cDNA using Superscript® III reverse transcriptase kit (Life technologies, USA). BIORAD CFX96 Touch^TM^ real-time machine was used for RT-PCR analysis using SYBR green PCR master mix (Applied Biosystems). Comparative *C*_*T*_ method (2^−ΔΔ*CT*^) was used for calculation of relative gene expression. Actin was used as endogenous control. Primers used for qRT-PCR assays are as follows: PKM2 (exon 10); forward 5′-TGCAATTATTTGAGGAACTCC-3′, reverse 5′-CACTGCAGCACTTGAAGGAG-3′. HKII; forward 5′- CCAACCTTAGGCTTGCCATT -3′, reverse 5′- CTTGGACATGGGATGGGGTG-3′. GLUT 1; forward 5′- CTTTGTGGCCTTCTTTGAAGT-3′, reverse 5′- CCACACAGTTGCTCCACAT-3′. ACTIN; forward 5′- ACTCTTCCAGCCTTCCTTC-3′, reverse 5′′- ATCTCCTTCTGCATCCTGTC-3′.

### Stable shRNA silencing and transient transfections

For stable knockdown of PKM2: the lentiviral particles were generated as described^63^. Briefly, HEK293T cells were transfected with transfer vector (LKO.1) containing shRNAs, plus packaging vectors- psPAX and pMD2.G, using Lipofectamine® 3000 (ThermoFisher Scientific, USA). Forty eight hours post-transfection, viral particles were harvested and used to infect the H1299 cells. Selection of infected cells was done in DMEM containing 2 µg/ml puromycin over the period of 2 weeks. For PKM2 over-expression: pcDNA-PKM2-myc or pcDNA-myc were transfected using Lipofectamine® 3000 reagent (ThermoFisher Scientific, USA), as per the manufacturer’s instructions.

### MTT assay for cell viability

Cell viability was assessed using MTT (3-(4, 5-Dimethylthiazol-2yl)-2, 5-diphenyl tetrozolium bromide) (Sigma). Exponentially growing cells were seeded in 96 well plate with the density of 10,000 cells per well. Subsequent to the cell adherence to the surface of culture plate (after approximately 12 hours), cells were treated with 20 μM curcumin. Cell viability was measured every 24-hour interval (24 to 72 hours) by washing the cells and replacing with fresh medium containing 20 μl of MTT (5 mg/ml in PBS) to each well. The plates were incubated for three hours in dark. The formazon crystals developed were solubilized with 100 μl of DMSO and the plate was kept in dark for another 5–10 min. Absorbance was measured using a microplate reader (Molecular Device) at 570 nm. Cells were seeded in triplicates for each group and the experiment was independently repeated thrice.

### Statistical analysis

Data were represented as mean with SD. Student’s t-test or one-way or two-way ANOVA with multiple comparisons was used to calculate statistical significance. *P* < 0.05 was considered to be statistically significant. Statistical significance is represented as: *P < 0.05; **P < 0.01; ***P < 0.001.

## Electronic supplementary material

Supplementary Information
