# Supplementary material for: Curcumin decreases Warburg effect in cancer cells by down-regulating pyruvate kinase M2 via mTOR-HIF1α inhibition
Source: Sci Rep. 2018 May 29;8:8323. doi: 10.1038/s41598-018-25524-3 (PMC5974195; doi:10.1038/s41598-018-25524-3)

Curcumin decreases Warburg effect in cancer cells by down-regulating pyruvate kinase M2 via mTOR-HIF1α inhibition.

Farid Ahmad Siddiqui[1], Gopinath Prakasam [2], Shilpi Chattopadhyay[3], Asad Ur Rehman[4], Rayees Ahmad Padder[1], Mohammad Afaque Ansari[1], Rasha Irshad[1], Kailash Mangalhara[2], Rameshwar NK Bamezai[2], Mohammad Husain[1], Syed Mansoor Ali[1] and Mohammad Askandar Iqbal[1†.]

[1] Department of Biotechnology, Faculty of Natural Sciences, Jamia Millia Islamia, 1 110025, India

[2] School of Life Sciences, Jawaharlal Nehru University, New Delhi 110067, India

[3] Department of Toxicology, Jamia Hamdard University, New Delhi 110062, India

[4.] Department of Biosciences, Faculty of Natural Sciences, Jamia Millia Islamia, New Delhi 110025, India

[†] Corresponding author: askandariqbal@gmail.com, miqbal2@jmi.ac.in.

Supplementary Figure S1.

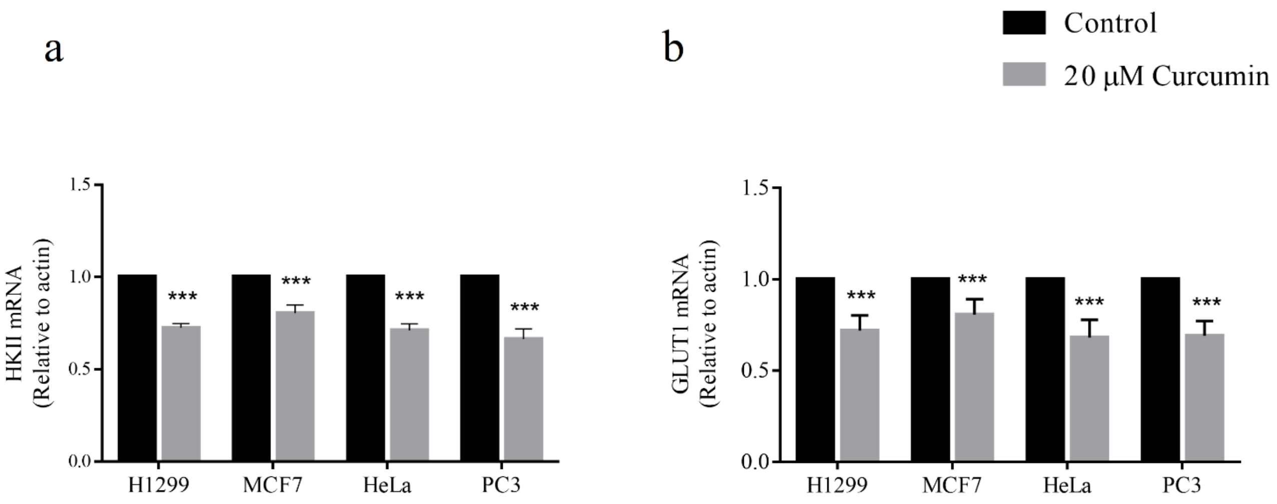

Supplementary Figure S1. HKII and GLUT1 expression is inhibited by curcumin through mTOR/HIF1α axis. Substantial reduction in HKII (a) and GLUT1 mRNA (b) in H1299, MCF-7, HeLa and PC3 cells treated with 20 µM of curcumin for 24 hours. Error bars in HKII and GLUT1 mRNA graph represent mean ±SD.

Supplementary Figure S2.

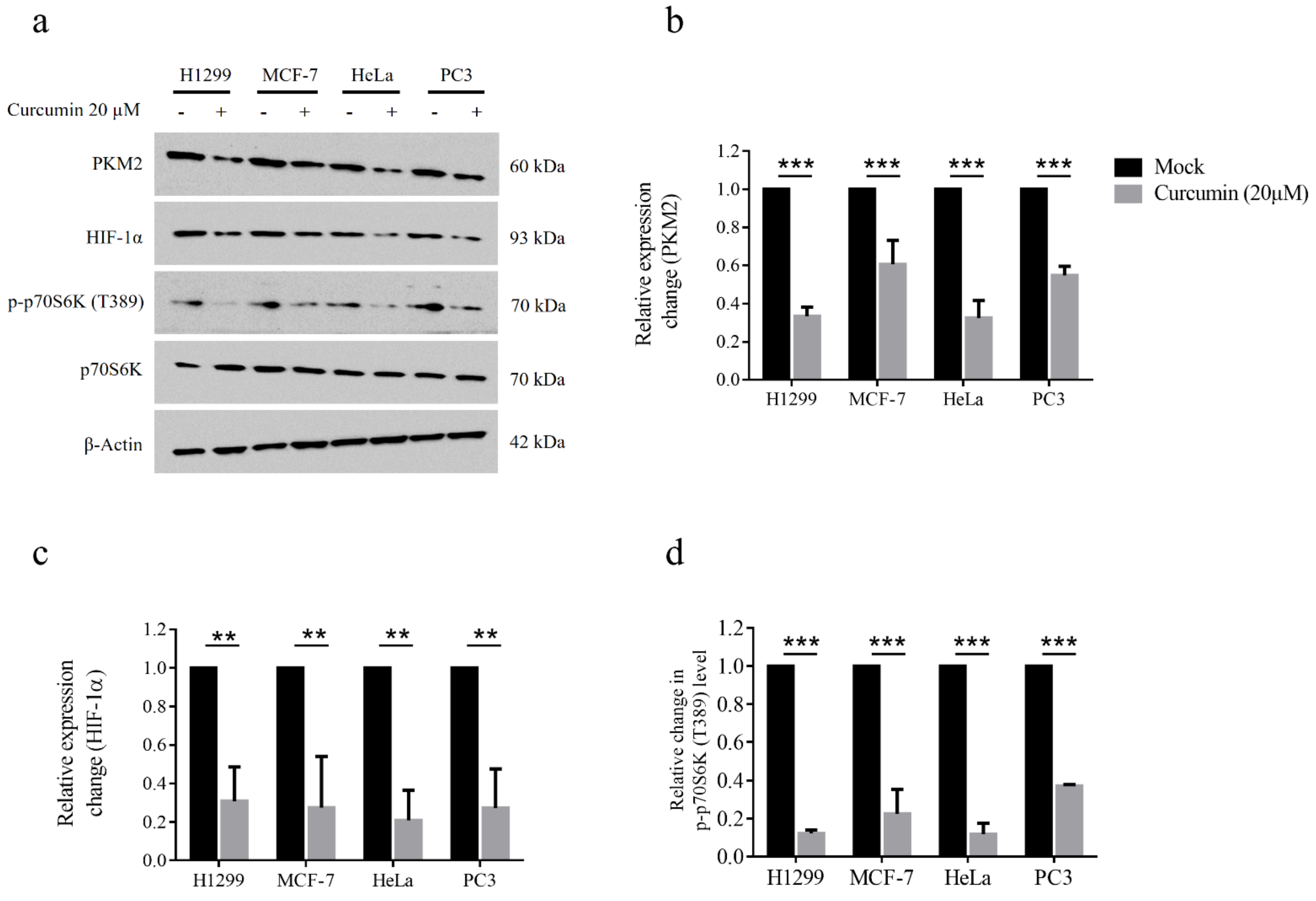

Supplementary Figure S2. PKM2 expression is inhibited by curcumin through mTOR/HIF1α axis. (a) Immunoblot of H1299, MCF-7, HeLa and PC3 cells showing coincided reduction in PKM2, HIF1α and phosphorylated-p70S6K (T389), upon curcumin treatment. Bars represent the relative expression level of PKM2 (b), HIF1α (c) and phosphorylated-p70S6K (T389) (d) in H1299, MCF-7, HeLa and PC3 cells treated with 20μM of curcumin for 24 hours. Error bars in graph represent mean ±SD.

Full-length gels uncropped Western Blots

Figure 2B Raw Data

PKM2
60kDa

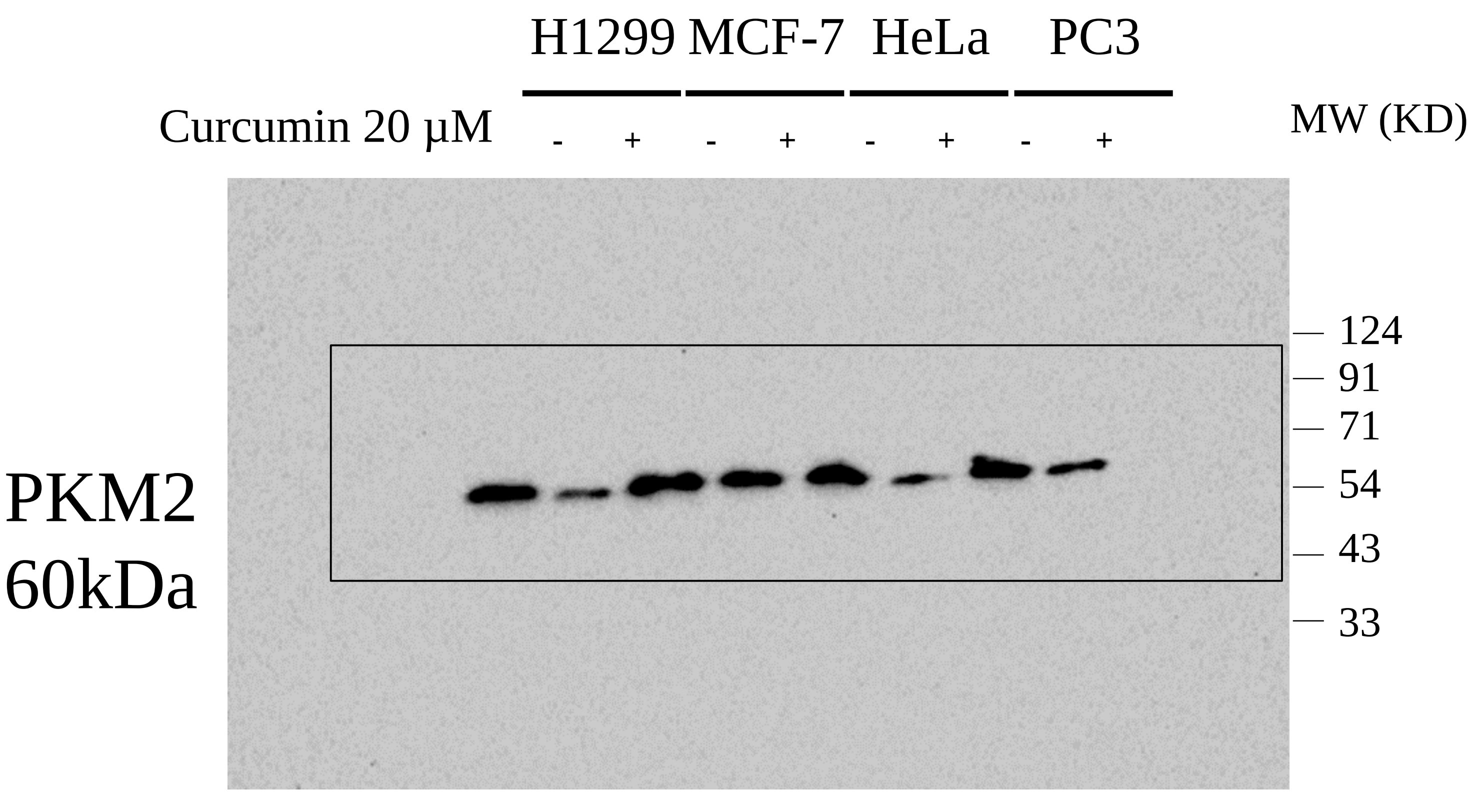

HIF-1 alpha
93 kDa

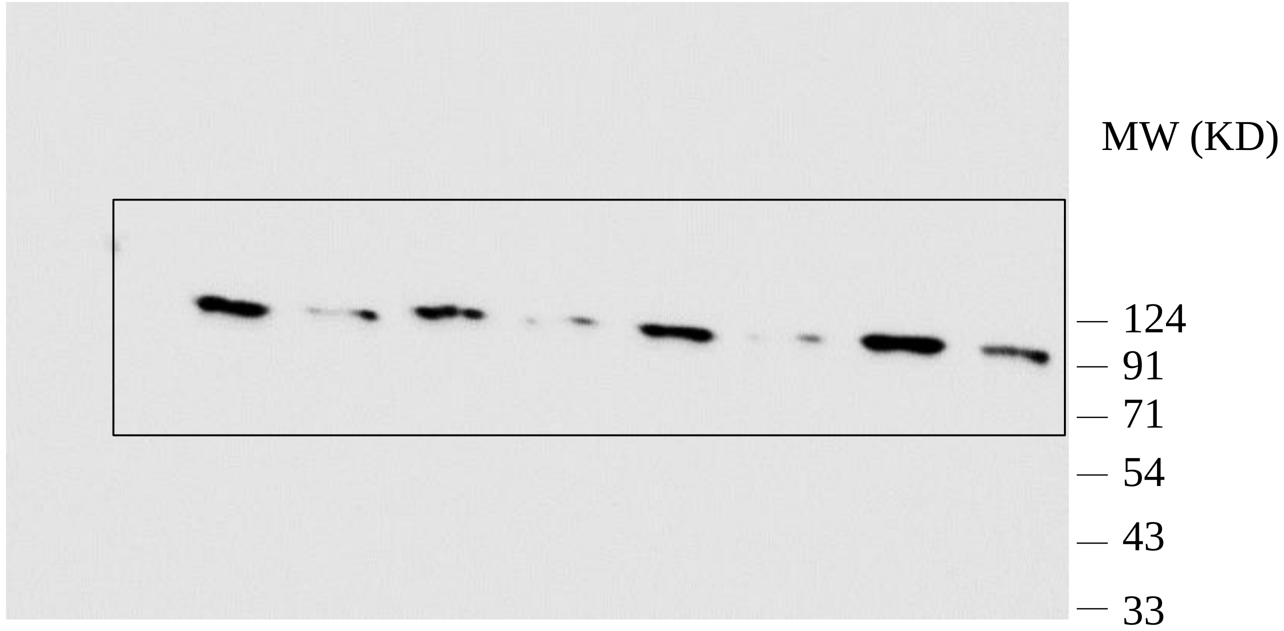

p-p70S6K(T389)
70 kDa

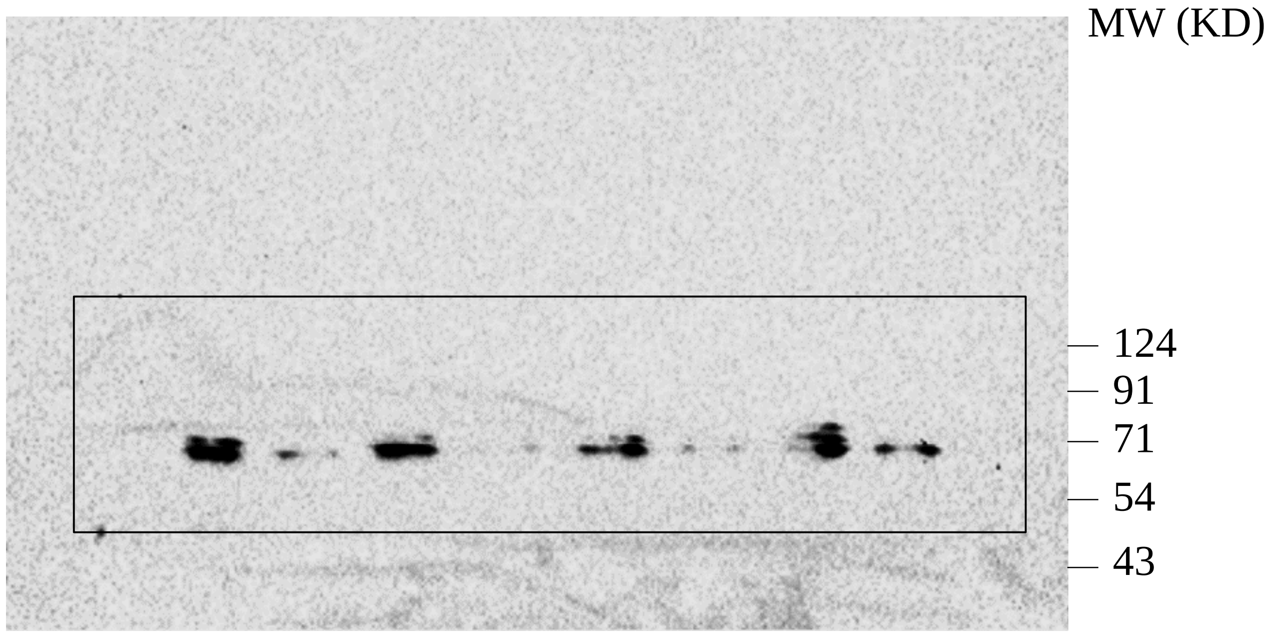

Figure 2B Raw Data Cont..

	H1299		MCF-7		HeLa		PC3	
Curcumin 20 µM	-	+	-	+	-	+	-	+

p70S6K
70 kDa

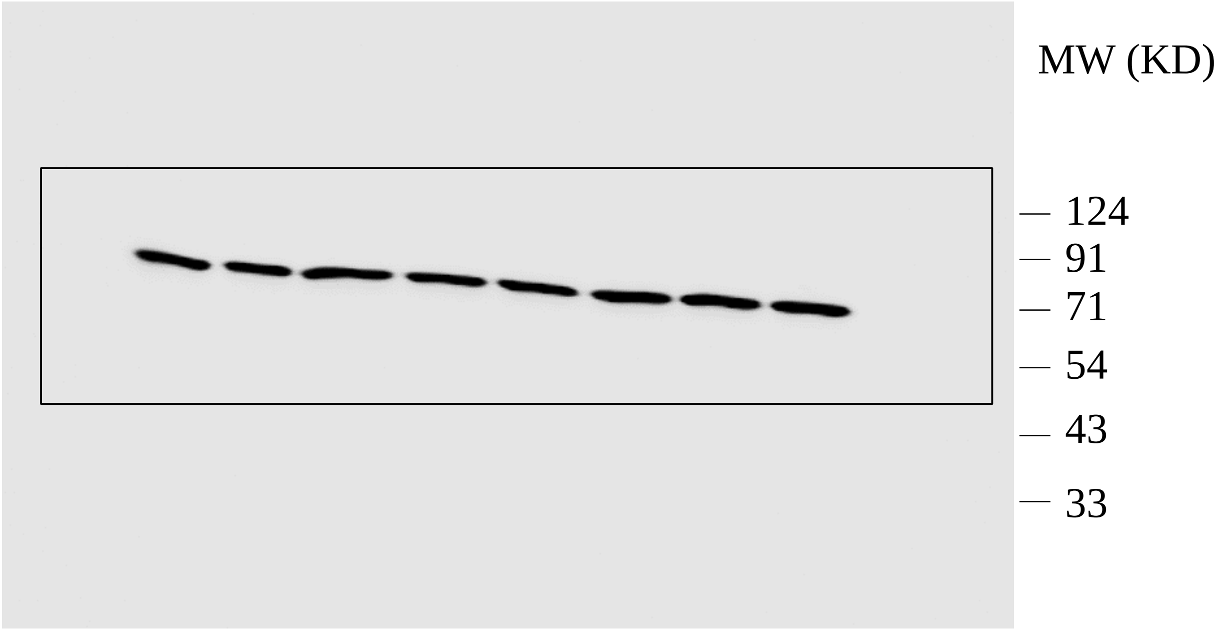

β-Actin
42 kDa

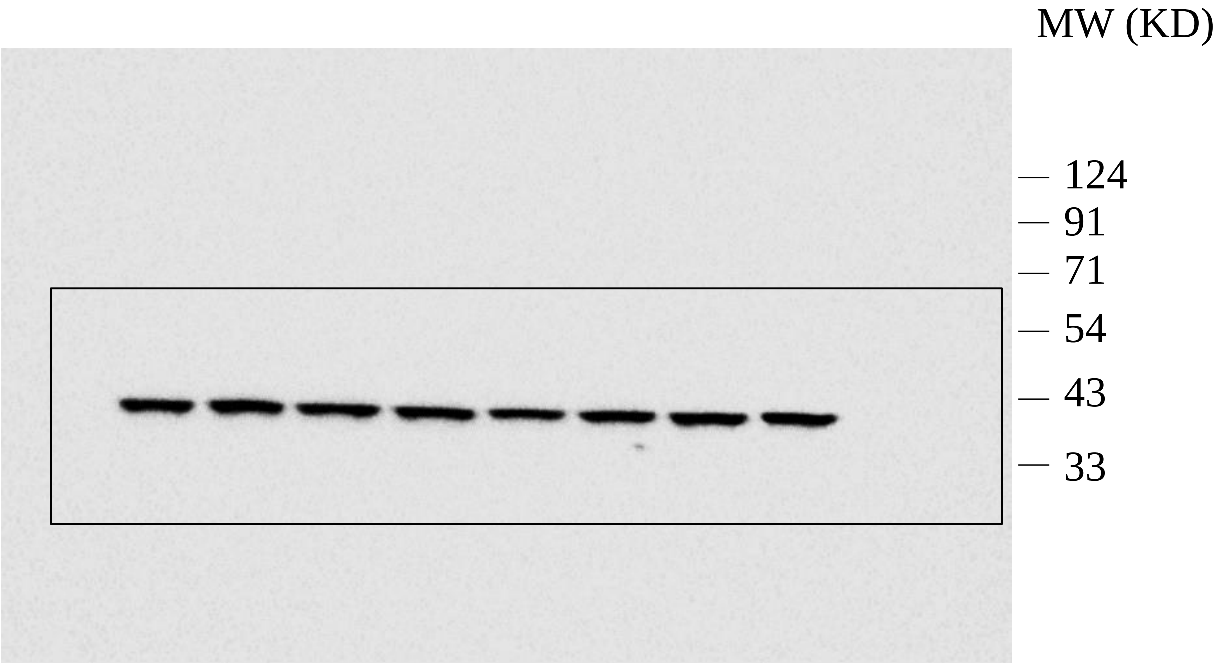

Figure 2C Raw Data

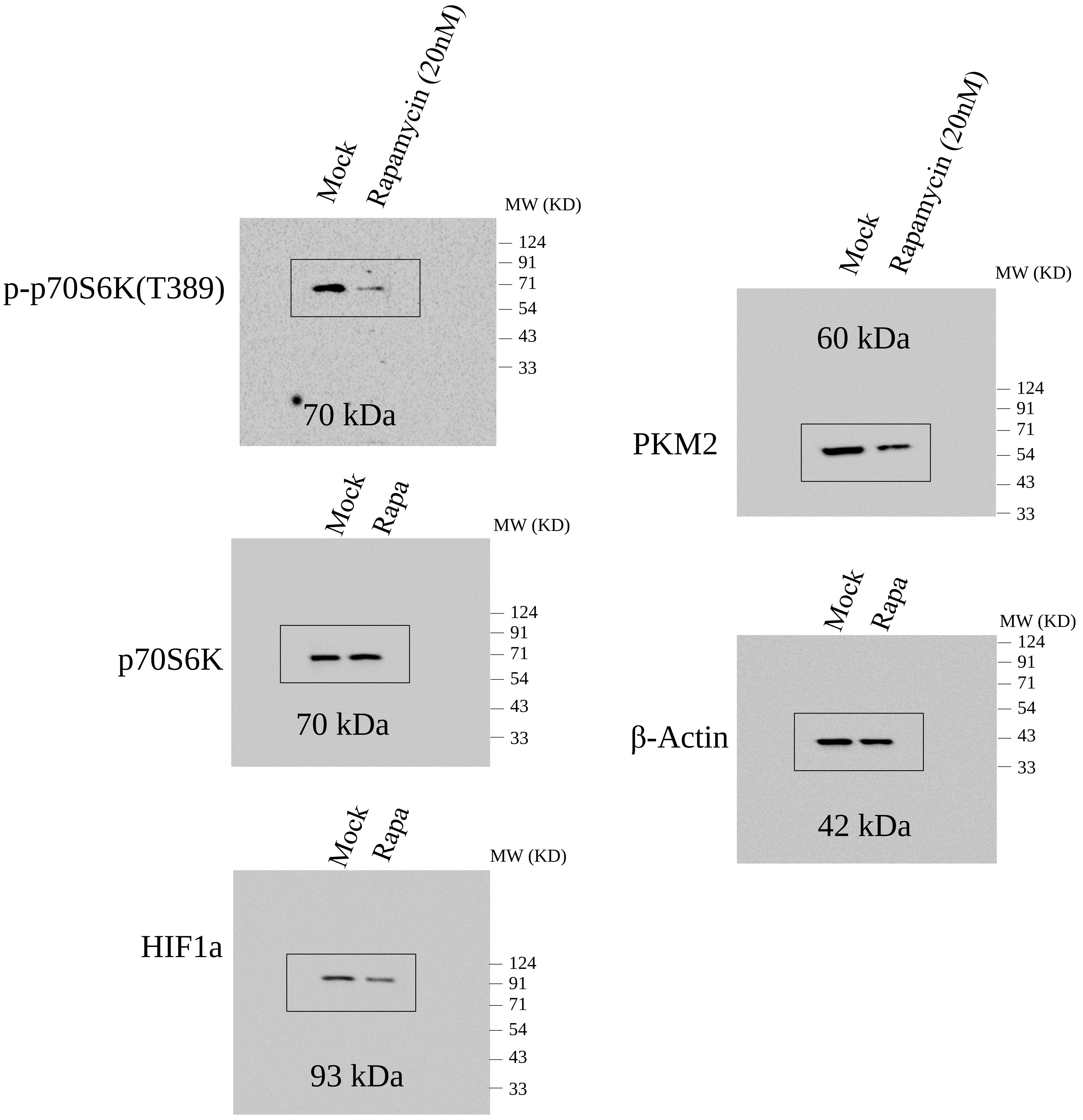

Figure 4A Raw Data

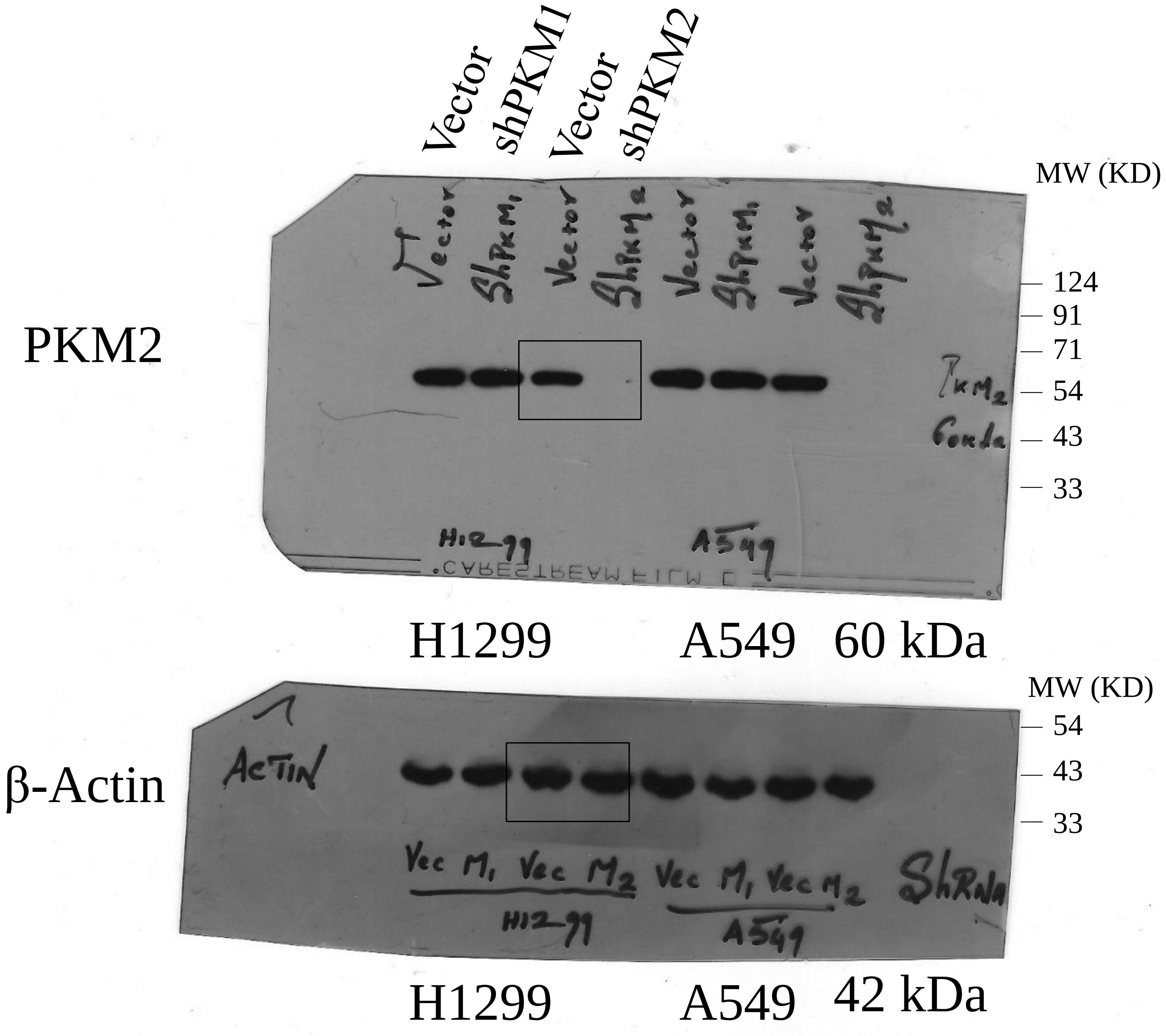

Figure 5A Raw Data

Myc-tag
60 kDa

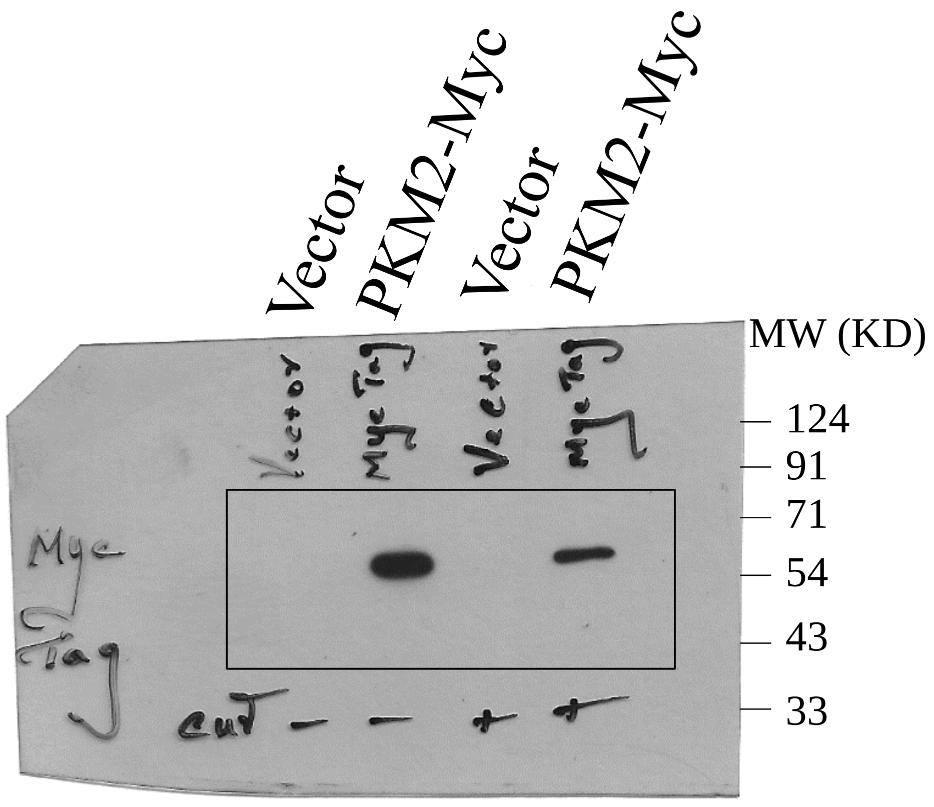

p-p70S6K(T389)
70 kDa

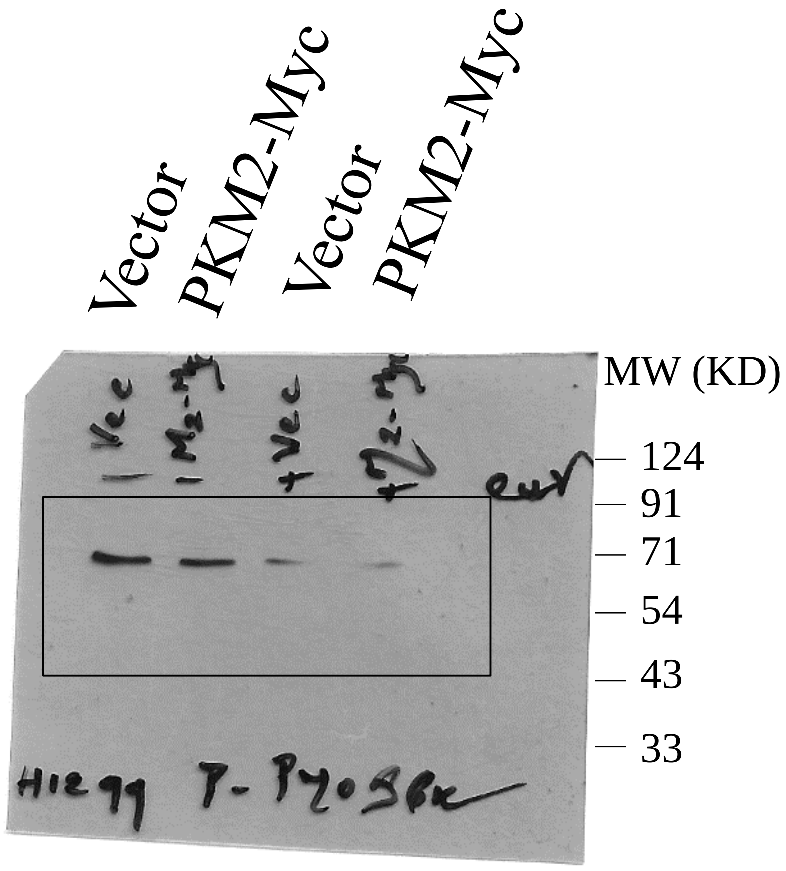

p70S6K
70 kDa

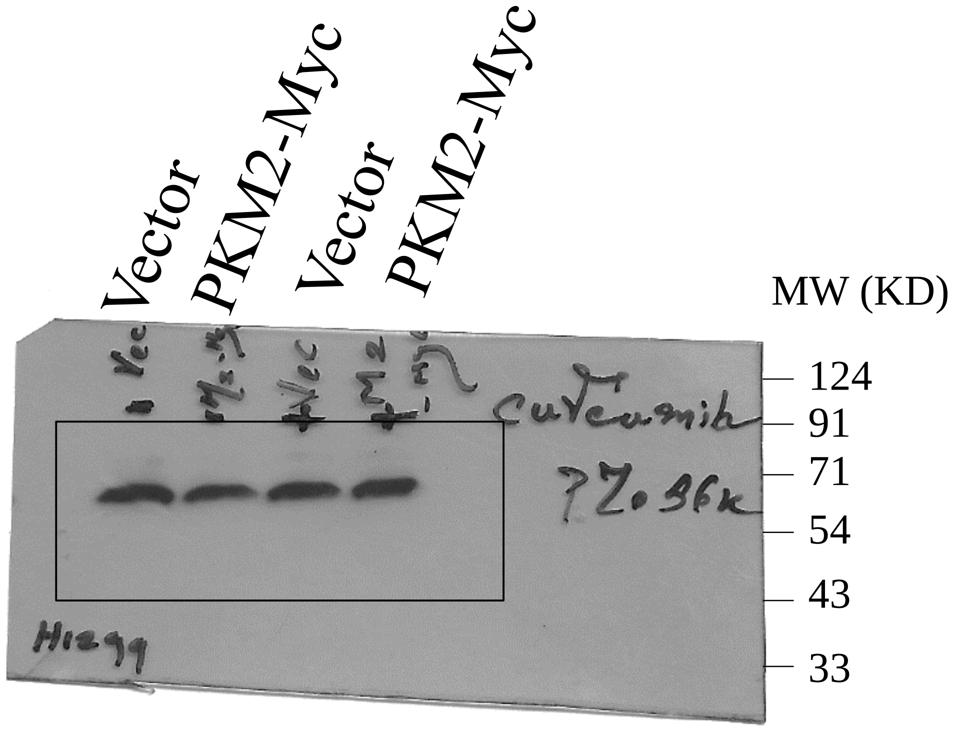

β-Actin
42 kDa

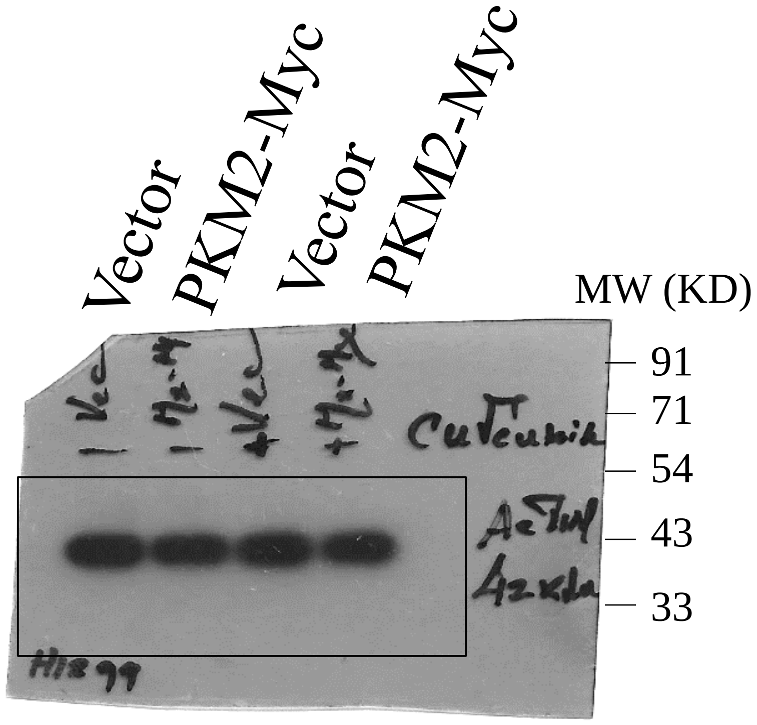

Supplementary Figure 2 Raw data

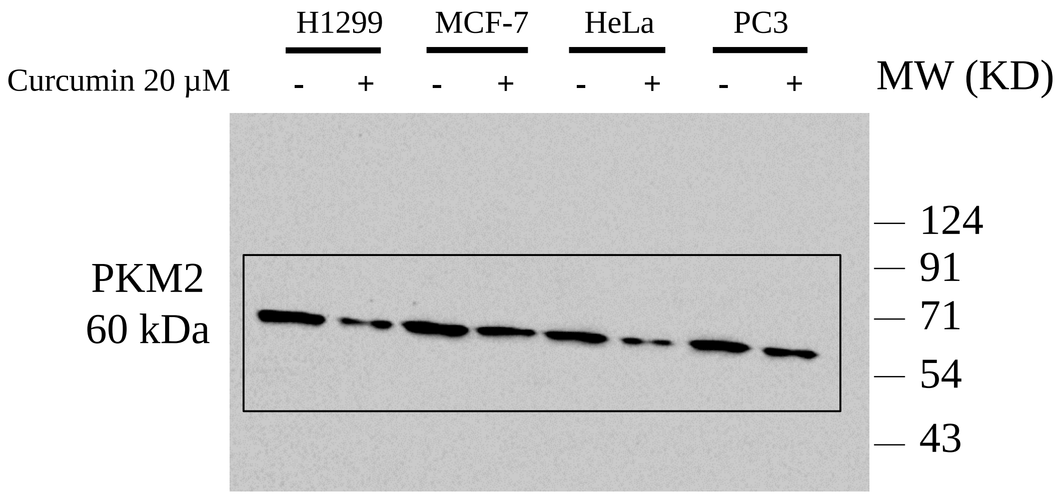

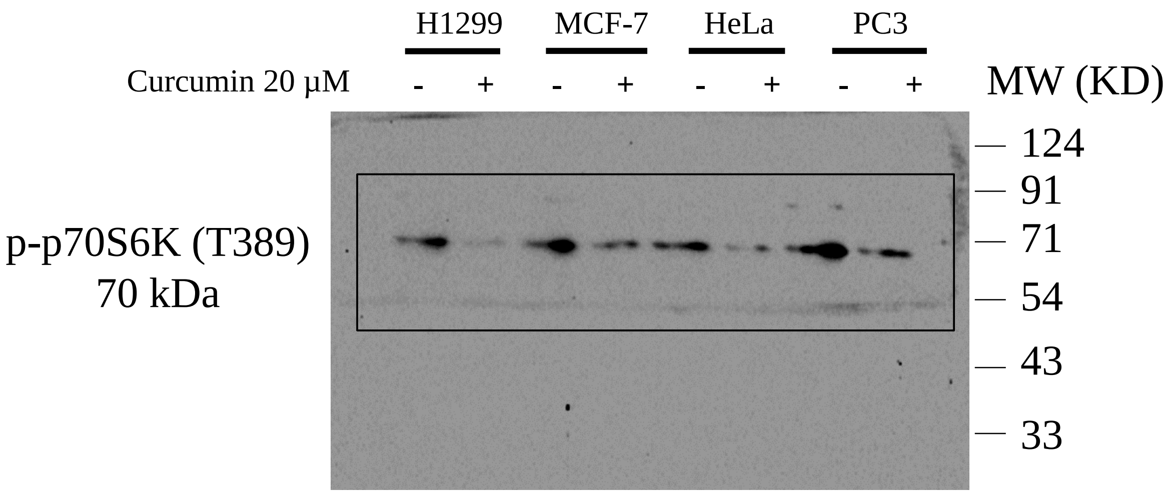

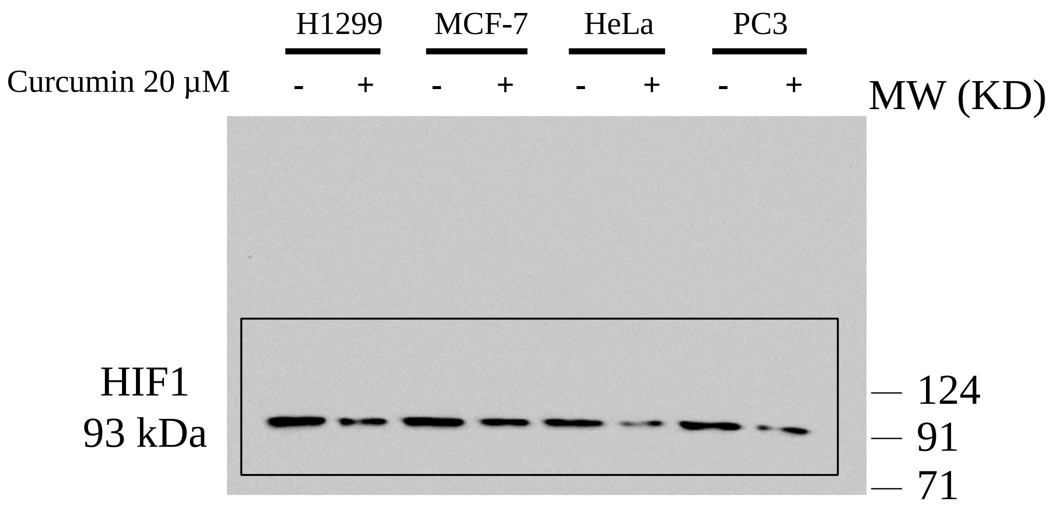

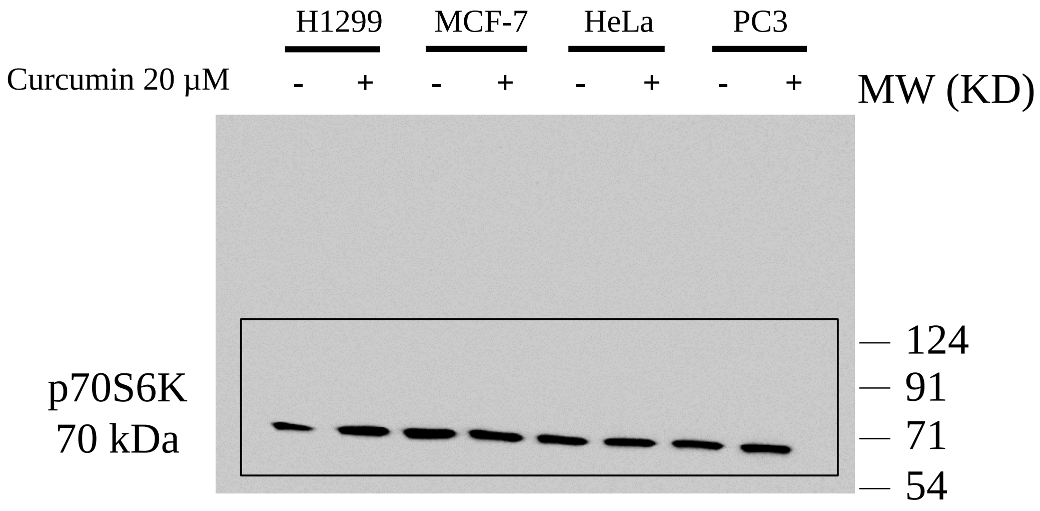

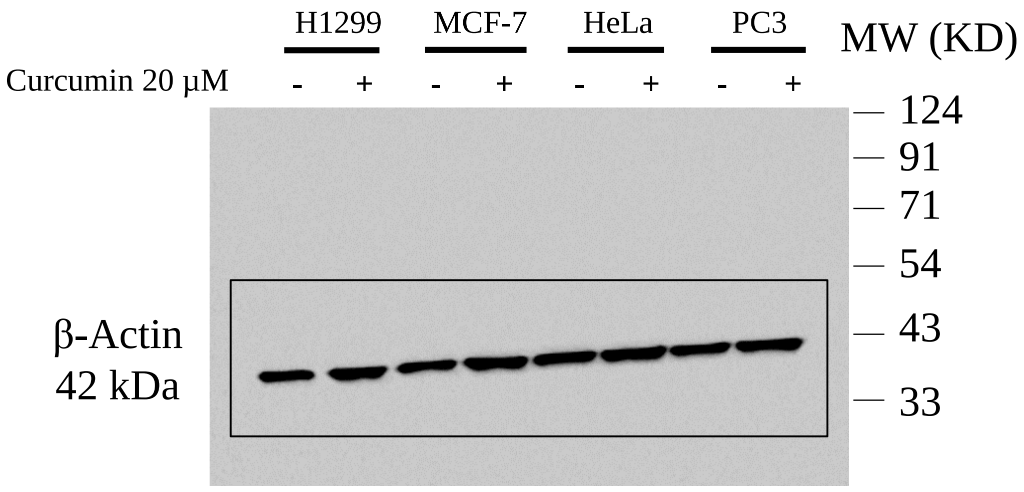

Supplement: Supplementary file 1 — Supplementary Information [file 41598_2018_25524_MOESM1_ESM.pdf]
